# Fenbendazole Exhibits Differential Anticancer Effects In Vitro and In Vivo in Models of Mouse Lymphoma

**DOI:** 10.3390/cimb45110560

**Published:** 2023-11-08

**Authors:** Haebeen Jung, Si-Yeon Kim, Hong-Gu Joo

**Affiliations:** 1 College of Veterinary Medicine, Jeju National University, Jeju 63243, Republic of Korea; 2 Veterinary Medical Research Institute, Jeju National University, Jeju 63243, Republic of Korea

**Keywords:** fenbendazole, EL-4 cells, lymphoma, anticancer effect, tumor microenvironment

## Abstract

Fenbendazole (FBZ) has been safely used as an antiparasitic agent in animals for decades, and the anticancer effects of FBZ have been studied through various mechanisms. However, there is a lack of in vivo studies that include lymphoma. Therefore, this study examined the effects of FBZ on EL-4 cells and a mouse T lymphoma model. FBZ induced G2/M phase arrest in EL-4 cells, resulting in cell death and decreased metabolic activity. However, FBZ had no anticancer effects on an EL-4 mouse lymphoma model in vivo, as evident by rapid weight loss and tumor growth comparable to the control. The FBZ-treated EL-4 cells expressed higher levels of PD-L1 and CD86, which are associated with T cell immunity in the tumor microenvironment (TME), than the controls. Furthermore, the hematoxylin and eosin staining of the FBZ-treated tumor tissues showed a starry sky pattern, which is seen in actively proliferating cancer tissues, and an immunohistochemical analysis revealed a high percentage of immunosuppressive M2 macrophages. These changes in the immune activity in the TME contradict the results of the in vitro experiments, and further studies are needed to determine the detailed mechanisms by which FBZ induces these responses.

## 1. Introduction

Fenbendazole (FBZ) is a benzimidazole anthelmintic agent that has been used safely for decades [1], and it is commonly used in animals, including livestock [2], companion animals [3], and laboratory animals [4]. FBZ binds more strongly to β-tubulin in parasitic cells than in mammals, inhibiting the formation of microtubules and causing parasite death [5]. This mechanism is similar to that of anticancer drugs targeting microtubules in cancer cells, such as paclitaxel and vinca alkaloids such as vincristine and vinblastine [6]. Studies have shown that FBZ exerts anticancer effects by inhibiting reactive oxygen species (ROS) production in cancer cells [7,8], arresting the G2/M phase [9,10], and impairing glucose metabolism [11]. Furthermore, the interest in FBZ has been fueled by a report of anticancer effects of FBZ in an American patient [12].

Statistics from the American Cancer Society published in January 2023 revealed that the incidence and mortality rates of lymphoma are among the highest, with non-Hodgkin’s lymphoma having a high incidence in young adults, ranking in the top 10 for both incidence and mortality [13,14]. Lymphoma is among the most commonly diagnosed cancers in dogs [15]. The first-line chemotherapy for lymphoma in humans and dogs is CHOP, which is a sequential combination of cyclophosphamide (CPA), doxorubicin (DOX), vincristine (VC), and prednisone (PDS) [16]. However, 30–40% of CHOP-treated patients do not respond to treatment or subsequently relapse [17], with relapse occurring more commonly in dogs [18]. Various chemotherapies and immunotherapies are used after CHOP treatment in relapsed patients, and further research is required to minimize chemoresistance and side effects [19].

As of 2023, there is a dearth of conclusive experimental data despite the interest in the anticancer effects of FBZ [20]. Notably, FBZ has not been studied in vivo in most cancers, including lymphoma. Therefore, in this study, we investigated the effects of FBZ on EL-4 cells in vitro and in vivo, using CHOP as a positive control. EL-4 cells were treated with FBZ, and their metabolic activity, cell death, cell cycle, ROS production, and mitochondrial membrane potential (MMP) were analyzed. The effects of FBZ on an EL-4-cell-induced mouse lymphoma model were investigated.

## 2. Materials and Methods

### 2.1. Animals and Reagents

C57BL/6 mice were purchased from Samtaco Bio Korea (Osan, Republic of Korea) and housed in the animal facility at Jeju National University. In this study, 7−10-week-old mice were used, and all animal experiments were performed in accordance with the Institutional Guidelines for Animal Use and Care of Jeju National University (No.: 2022-0068). Fenbendazole was purchased from Sigma-Aldrich (St. Louis, MO, USA) and dissolved in dimethyl sulfoxide (DMSO; Sigma-Aldrich). CPA (Tokyo Chemical Industry, Tokyo, Japan), DOX (Teva-Handok, Seoul, Republic of Korea), VC (REYON Pharmaceutical Co., Seoul, Republic of Korea) and PDS (Sigma-Aldrich) were used as positive controls. CHOP (100 µM) indicates a mixture of CPA (100 µM), DOX (5 µM), VC (0.14 µM), and PDS (10 µM) [21], and the same ratio was used for this study (1 mg/mL CHOP; a mixture of 1 mg/mL of CPA, 0.1 mg/mL of DOX, 0.005 mg/mL of VC, and 0.14 mg/mL of PDS).

### 2.2. Cell Lines

EL-4 (mouse lymphoma cell line) was purchased from Korean Cell Line Bank (Seoul, Republic of Korea). The cells were cultured in RPMI 1640 medium containing 10% fetal bovine serum, 100 U/mL penicillin-streptomycin, and 2 mM L-glutamine.

### 2.3. Measurement of Cellular Metabolic Activity

To measure the metabolic activity of the cells, EL-4 cells (1 × 10^5^ cells/mL) were treated with FBZ or CHOP and cultured in 96-well plates for 3 days. 3-(4,5-di methylthiazol-2-yl)-2,5-diphenyltetrazoliumbromide (MTT, Sigma-Aldrich) solution was added at a concentration of 0.5 mg/mL and incubated at 37 °C in 5% CO_2_ for 4 h. A 10% sodium dodecyl sulfate solution was added to dissolve the formazan product (crystal violet) produced by the cells and reacted for 2 h. Optical density was measured at 570 nm using a microplate reader (Multiskan FC; Thermo Fisher Scientific, Waltham, MA, USA).

### 2.4. Nuclear Staining for Morphological Assessment of Cell Death

For the observation of cell nuclei appearance and to analyze cell death, Hoechst 33342 (Hoechst; Sigma-Aldrich) and propidium iodide (PI; Sigma-Aldrich) were double-stained to EL-4 cells. EL-4 cells (1 × 10^5^ cells/mL) were treated with FBZ or CHOP and incubated for 3 days. Cells were then stained with Hoechst 33342 solution and PI solution at a concentration of 2.5 μg/mL each for 10 min at 37 °C. A fluorescence microscope (ZOE Fluorescent Cell Imager; BIO-RAD, Hercules, CA, USA) was used for observation.

### 2.5. Flow Cytometry Analysis

EL-4 cells (1 × 10^5^ cells/mL) were treated with FBZ or CHOP in 24-well culture plates. The expressions of PD-L1, FasL, CD86, ROS production, and MMPs were measured after 16 h, and annexin V/PI staining and cell cycle analysis were performed after 48 h. Cells were washed with FACS staining solution (5% FBS and 0.1% sodium azide in Hanks’ Balanced Salt Solution, FSS) and treated with primary antibodies (Biotin labeled I-Ab, PD-L1, FasL, and CD86 mAb; BioLegend, San Diego, CA, USA) followed by streptavidin-conjugated allophycocyanin (BioLegend) as secondary antibodies. TruStain FcXTM (anti-mouse CD16/32; clone 93; BioLegend) was used as an Fc blocker to prevent non-specific reactions. For ROS analysis, 2′,7′-dichlorofluorescin diacetate (DCFDA; Sigma-Adrich) solution was used at a concentration of 5 μM for 30 min at 37 °C. For the measurement of MMPs, Rhodamine 123 (Sigma-Aldrich) solution was used at a concentration of 0.25 μg/mL and reacted for 30 min at room temperature in the dark. For cell cycle analysis, DNA was stained with PI. Cells were washed with phosphate-buffered saline (PBS) and fixed with ethanol for 20 min at −20 °C. Cells were treated with 0.2 mg/mL RNase for 30 min and then stained with 50 μg/mL PI. For annexin V/PI staining, cells were treated with annexin V-fluorescein isothiocyanate (FITC) (BD Bioscience, Franklin Lakes, NJ, USA) and PI solution at a concentration of 5 µL/mL and 1.5 µg/mL, respectively, and reacted for 10 min at room temperature in the dark. After all reactions, except PI staining, cells were washed twice with FSS. Stained cells were flow cytometrically analyzed using a CytoFLEX LX flow cytometer (Beckman Coulter, Brea, CA, USA) and CytExpert 2.5 software (Beckman Coulter).

### 2.6. In Vivo Study and Drug Treatment

To ensure a consistent tumor model, C57BL/6 mice were injected subcutaneously (SC) with 3 × 10^6^ EL-4 cells for a single in vivo passage. Tumor-derived EL-4 (tmEL-4) cells were harvested and 1 × 10^6^ tmEL-4 cells were injected by SC into the right flank of 7–10-week-old female C57BL/6 mice. Body weight and tumor size were measured daily. Tumor size was measured with Vernier calipers, and the volume of the tumor was converted to length × width^2^ × 0.5 (mm^3^). Mice were randomly divided into three groups (*n* = 6 or 7) when their tumors reached approximately 100 mm^3^, and day 0 was set. On days 0, 3, and 6, drugs were inoculated intraperitoneally (IP) as follows: vehicle control group (PBS 120 μL + DMSO 20 μL), CHOP-treated group (CHOP 40 mg/kg), and FBZ-treated group (25 mg/kg). Mice were sacrificed on day 10 and tumor tissues were harvested. Tumor tissues were weighed and fixed in 4% paraformaldehyde solution for tissue specimen preparation.

### 2.7. Histology

Fixed tissues were dehydrated and cleared with ethanol and xylene, embedded in paraffin, and sectioned at 5 μm thickness. Tissue sections were stained with hematoxylin and eosin (H&E) or immunohistochemistry (IHC), and then microscopically observed.

### 2.8. Immunohistochemistry (IHC)

IHC was performed using the VECTASTAIN Elite ABC-Peroxidase Kit (Vector Laboratories, Newark, CA, USA). Deparaffinized tissue sections were immersed in 0.3% hydrogen peroxide to remove endogenous peroxidase. To prevent non-specific immunoreactivity, goat serum was reacted for 45 min, followed by primary antibodies for 16 h at 4 °C with conditions shown in Table 1. Subsequently, biotinylated anti-rabbit IgG (Vector Laboratories) and avidin–biotin peroxidase conjugate (Vector Laboratories) were reacted for 45 min each at room temperature. Immunoreacted tissues were developed with 3,3’-diaminobenzidine (DAB; Vector Laboratories) solution, and the reaction was terminated in distilled water. Three 5 min washes with PBS were performed between each step. Counter staining was performed with hematoxylin, and then tissues were dehydrated, cleared, and embedded. Three to five fields per mouse were analyzed using Image J v1.54 (National Institute of Health, Bethesda, MD, USA) at 100× magnification.

### 2.9. Statistical Analysis

All experiments were repeated at least three times under the same conditions and statistically analyzed. Data were shown as mean ± standard deviation, and significance was determined using Dunnett’s multiple comparisons test (GraphPad Prism 9 Software, USA) after one-way or two-way ANOVA analysis. NS indicates non-significance. *, **, and *** indicate *p* < 0.05, 0.01, and 0.001 compared to the control.

## 3. Results

### 3.1. FBZ and CHOP Decreased the Metabolic Activity of EL-4 Cells

**Figure 1:**
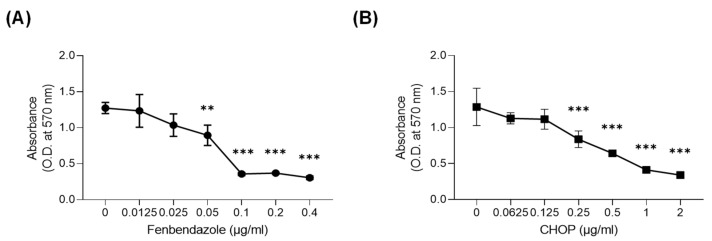
FBZ and CHOP decreased the metabolic activity of EL-4 cells. Metabolic activity of EL-4 cells treated with FBZ (**A**) and CHOP (**B**) were investigated using an MTT assay. EL-4 cells were cultured in a 96-well plate at a concentration of 1 × 10^5^ cells/mL and incubated with FBZ or CHOP for 3 days. MTT solution and 10% SDS solution were added subsequently, and the optical density was measured at 570 nm using a microplate reader. Statistical significance was determined via one-way ANOVA, followed by Dunnett’s multiple comparisons test. ** and *** indicate *p* < 0.01 and 0.001 compared to the control (0 μg/mL).

To investigate the effects of FBZ and CHOP on the metabolic activity of EL-4 cells, an MTT assay was performed (Figure 1). When treated with 0.05 μg/mL FBZ and 0.25 μg/mL CHOP, the metabolic activity of the EL-4 cells significantly decreased. Based on the MTT assay results, the half-maximal inhibitory concentration (IC_50_) of each drug against EL-4 cells was 0.05 μg/mL for FBZ and 0.3 μg/mL for CHOP.

### 3.2. FBZ and CHOP Induce the Apoptosis and Necrosis of EL-4 Cells

**Figure 2:**
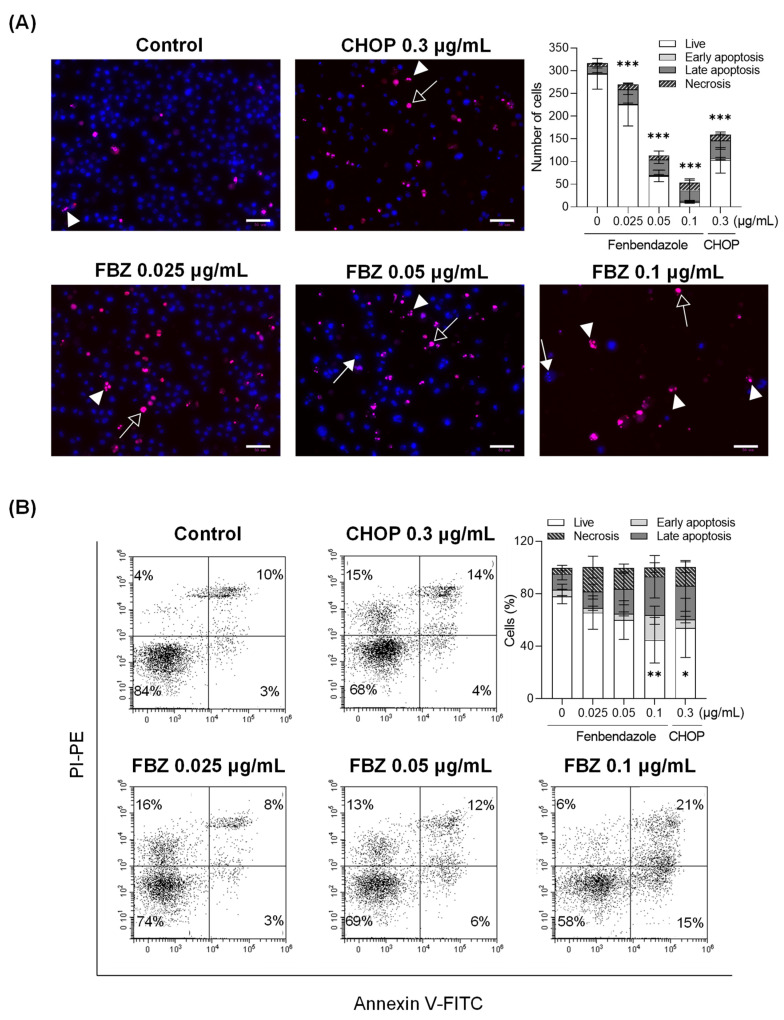
FBZ and CHOP induced the apoptosis and necrosis of EL-4 cells. For cell death analysis, EL-4 cells (2 × 10^5^ cells/mL) were treated with FBZ or CHOP. (**A**) After 72 h incubation, the cells were double-stained with Hoechst 33342 and PI, and the images were captured using a fluorescence microscope. Early apoptotic cells (arrows) are the cells with blue chromatin, which is highly condensed, marginated, or fragmented. Late apoptotic cells (arrow heads) indicate the cells with bright red condensed and fragmented chromatin. Necrotic cells (open arrows) are the cells with bright red or red enlarged nuclei with normal structures. Scale bar: 50 µm. (**B**) After 48 h incubation, annexin V/PI staining was performed. The quadrants of the dot plot indicate live cells (annexin V^−^/PI^−^) and cells in early apoptosis (annexin V^+^/PI^−^), late apoptosis (annexin V^+^/PI^+^), and necrosis (annexin V^−^/PI^+^). The graphs were generated using data from three independent experiments. Statistical significance was performed via two-way ANOVA, followed by Dunnett’s multiple comparisons test. *, **, and *** indicate *p* < 0.05, 0.01, and 0.001 of the live cells compared to the control (0 μg/mL).

Cell death in the EL-4 cells treated with FBZ and CHOP was analyzed in two ways. First, we morphologically observed the nuclear appearance via Hoechst 33342/PI staining and classified cell death as follows: early apoptosis, in which the nuclei begin to condense (condensed and segmented blue-stained nuclei; Hoechst^+^/PI^−^); late apoptosis, observed as condensed nuclei with damaged cell membranes (condensed and segmented pink-stained nuclei; Hoechst^+^/PI^+^); and necrosis, observed as damaged cell membranes and large or concentrated nuclei (pyknosis) (undivided pink- or red-stained nuclei; Hoechst^+/−^/PI^+^) (Figure 2A). Cell viability was reduced by 9%, 33%, and 73% at FBZ concentrations of 0.025 μg/mL, 0.05 μg/mL, and 0.1 μg/mL, respectively, and it was reduced by 28% at 0.3 μg/mL of CHOP compared to the control. In addition, the total cell number was reduced by 64%, 83%, and 50% when treated with 0.05 μg/mL of FBZ, 0.1 μg/mL of FBZ, and 0.3 μg/mL of CHOP, respectively, compared to the control.

When cells begin to undergo apoptosis, the phosphatidyl serine (PS) inside the cell is exposed to the outside through membrane changes. The exposed PS was stained with annexin V, and the cells with damaged membranes were double-stained with PI to observe cell death separately within the same number of cells (Figure 2B). When treated with 0.1 μg/mL of FBZ and 0.3 μg/mL of CHOP, the proportion of live cells (annexin V^−^/PI^−^) decreased by 33% and 24%, respectively, compared to that of the control, and the proportion of apoptotic cells (annexin V^+^/PI^+/−^) increased by 31% and 15%, respectively. When treated with 0.025 μg/mL of FBZ and 0.05 μg/mL of FBZ, the percentage of live cells decreased by 12% and 18%, and necrosis (annexin V^−^/PI^+^) was 14% and 11%, respectively, compared to the control.

### 3.3. FBZ Induced G2/M Phase Arrest While CHOP Produced Intracellular ROS in EL-4 Cells

**Figure 3:**
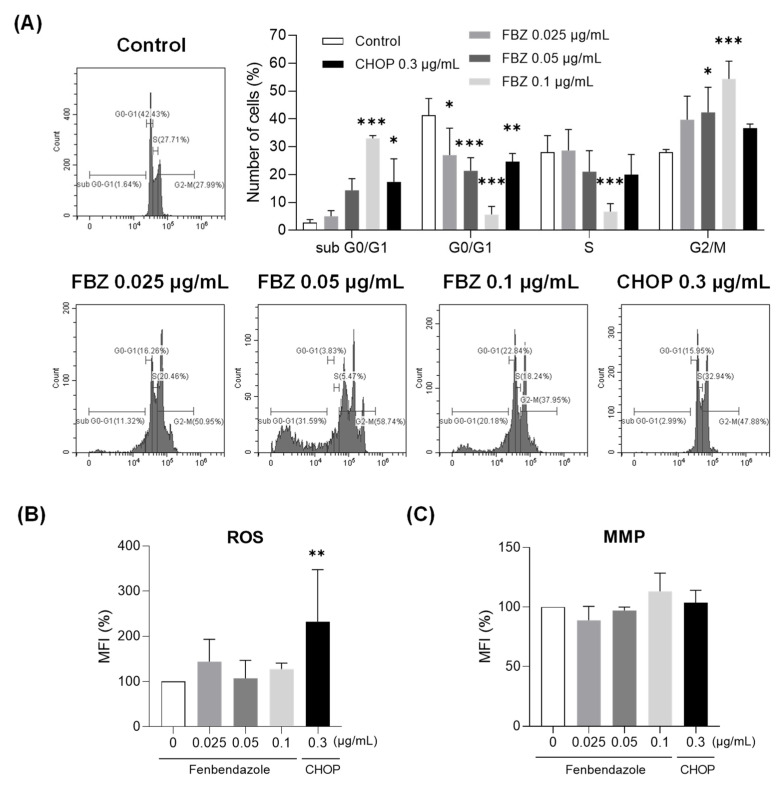
FBZ induced G2/M phase arrest while CHOP produced intracellular ROS in EL-4 cells. (**A**) EL-4 cells were treated with FBZ or CHOP for 48 h and stained with PI. Cell cycles of EL-4 cells were assessed via flow cytometry, and the percentage of cells in each phase is shown. To analyze intracellular ROS and MMP, EL-4 cells were treated with FBZ or CHOP for 16 h and stained with DCFDA (**B**) or Rhodamine 123 (**C**). For statistical analysis of three independent experiments, mean fluorescence intensity (MFI) of staining detected in the control EL-4 cells was set to 100%. Statistical significance was performed via one-way (**B**,**C**) or two-way (**A**) ANOVA, followed by Dunnett’s multiple comparisons test. *, **, and *** indicate *p* < 0.05, 0.01, and 0.001 compared to the control (0 μg/mL).

To determine whether FBZ and CHOP affected the cell cycle, DNA from the EL-4 cells treated with each drug was stained with PI (Figure 3A). When treated with 0.05 μg/mL of FBZ, the proportion of cells in the G2/M phase increased by 14% compared to the control. The number of cells in S phase decreased by 21% and the number of cells in G2/M phase increased by 26% when treated with 0.1 μg/mL FBZ compared to control cells. In other words, FBZ induced G2/M phase arrest, resulting in a significant decrease in the G0/G1 phase cells at all concentrations. When treated with 0.3 μg/mL of CHOP, the number of cells was reduced in the G0/G1 phase, but there was no significant change in the proportion of cells in the S and G2/M phases compared to the control cells.

The effects of FBZ and CHOP on intracellular ROS production and MMP were also investigated (Figure 3B,C). At concentrations near IC_50_, FBZ did not affect ROS production and MMP. CHOP increased intracellular ROS production at IC_50_ but did not alter the MMP.

### 3.4. Effects of FBZ and CHOP in EL-4 Cell-Derived Mouse Lymphoma Model

#### 3.4.1. Changes in Body Weight and Volume of Tumor Mass

**Figure 4:**
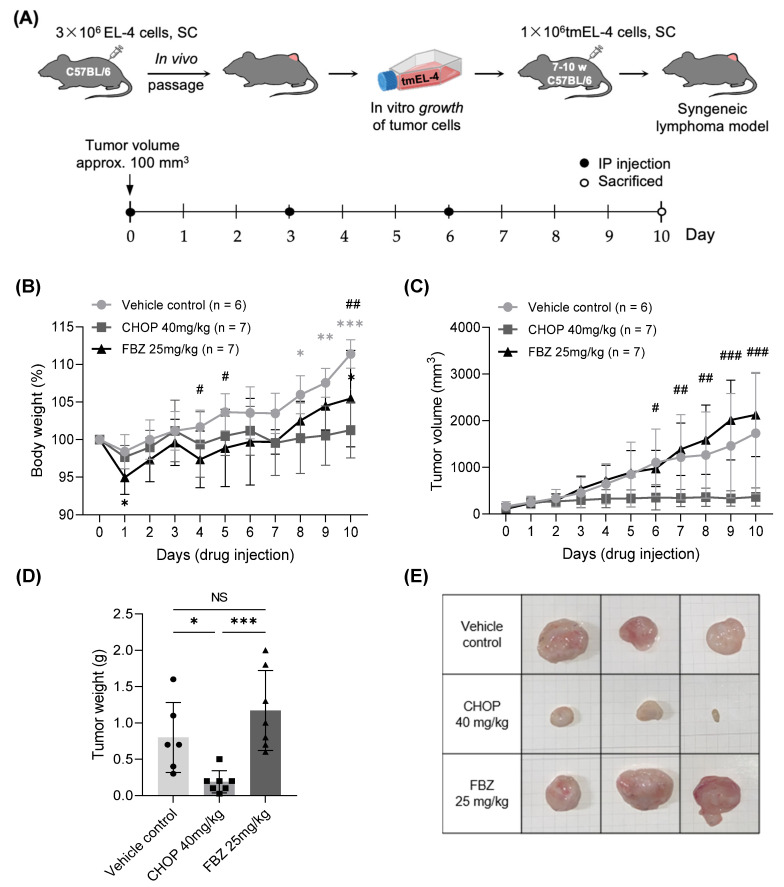
Body weight and tumor growth of EL-4 induced mouse lymphoma model. (**A**) After in vivo passage of EL-4 cells, tmEL-4 cells were harvested and injected subcutaneously into the right flank. Day 0 for treatment was defined as the day when the tumor reached a volume of approximately 100 mm^3^. Each group of mice was treated intraperitoneally with the following regimen: vehicle control (PBS 120 μL + DMSO 20 μL), CHOP (CHOP 40 mg/kg), or FBZ (FBZ 25 mg/kg). Body weight (**B**) and tumor volume (**C**) were checked daily. (**D**) On day 10, the mice were sacrificed, and the weights of tumors were determined. (**E**) Three representative photos of tumors in each group are shown. Scale bar: 1 cm. Statistical significance was performed via one-way ANOVA, followed by Dunnett’s multiple comparisons test. *, **, and *** indicate *p* < 0.05, 0.01, and 0.001 compared to day 0 (**B**) or CHOP-treated group (**D**). #, ##, and ### indicate *p* < 0.05, 0.01, and 0.001 between vehicle control and CHOP 40 mg/kg. NS: not significant.

To investigate the effects of FBZ and CHOP on lymphoma in vivo, a T cell lymphoma model was generated in mice using tmEL-4 cells, and the drugs were administered IP (Figure 4A). The control group showed a significant increase in the tumor volume from day 6 compared to day 0, with a corresponding increase of 6.0% in body weight on day 8 and a 11.4% increase on day 10 (Figure 4B,C). The FBZ-treated group showed a 5.0% decrease in body weight on day 1, and from day 5, despite the tumor volume increasing by ≥779.3 mm^3^ compared to that on day 0, the body weight showed no significant change; on day 10, the body weight increased by 5.5%. In the CHOP-treated group, neither the body weight nor tumor size changed compared to day 0. On day 6, the tumor volume of the CHOP-treated group was significantly smaller than that of the control group. There was no significant difference in the tumor weight between the control and FBZ-treated groups, and the tumor weight of the CHOP-treated group was 76.3% lower than that of the control group (Figure 4D,E).

#### 3.4.2. Histopathology

**Figure 5:**
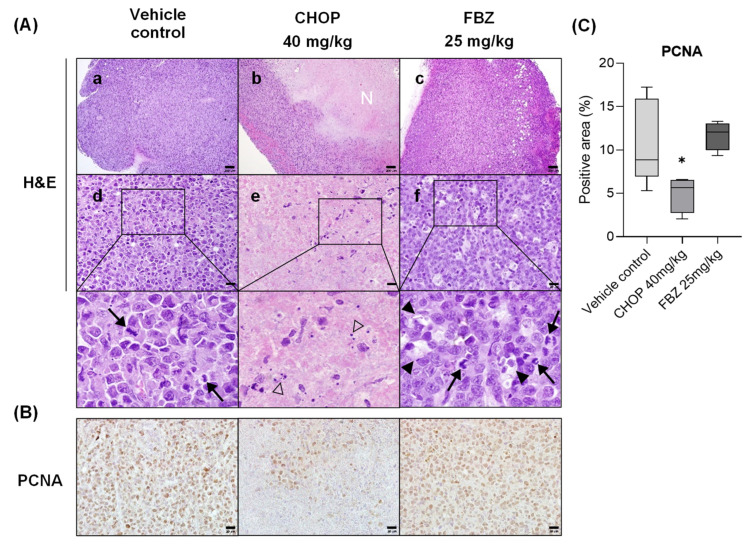
Effects of FBZ and CHOP on EL-4 lymphoma were assessed historically. (**A**) Mitotic index (arrows), tingible body macrophages (arrow heads), apoptotic bodies (open arrow heads), and necrosis region (N) were observed via H&E staining. Scale bar: 200 µm (**a**–**c**) and 20 µm (**d**–**f**). (**B**) PCNA staining was performed to confirm proliferative cells. Scale bar: 20 µm. (**C**) Average of the PCNA-positive area of 3–5 visual fields observed at 100× per mouse is graphically shown. Statistical significance was performed via one-way ANOVA, followed by Dunnett’s multiple comparisons test. * indicates *p* < 0.05 compared to the vehicle control.

EL-4 syngeneic lymphoma appeared as a unilateral subcutaneous infiltrating solid tissue at the injection site. The tumor tissues observed via H&E staining consisted of large densely packed and immature cells (Figure 5A). Many blood vessels were observed between the cancer cells and tissue margins. Mitotic figures were observed in the control and FBZ-treated groups under a high-power field (HPF; ×400 magnification), with a higher number observed in the FBZ-treated group (Figure 5(Ad,Af)). A starry sky pattern of tingible body macrophages (TBMs) was also observed in the tissues of the FBZ-treated group due to their high proliferation rate (Figure 5(Af)). The central part of the tumor tissue in the CHOP group was mostly composed of eosinophilic necrotic cells interspersed with apoptotic bodies, showing signs of cell death (Figure 5(Ab,Ae)).

PCNA staining was performed to determine the proliferation rate of the tumor tissues (Figure 5B,C). There was no significant difference in the proliferation rate between the control and FBZ-treated groups, but the proliferation rate of the CHOP-treated group was reduced by 53.2% compared with that of the control group.

### 3.5. FBZ Increased the Expression of PD-L1 and CD86 on EL-4 Cells but Not FasL

**Figure 6:**
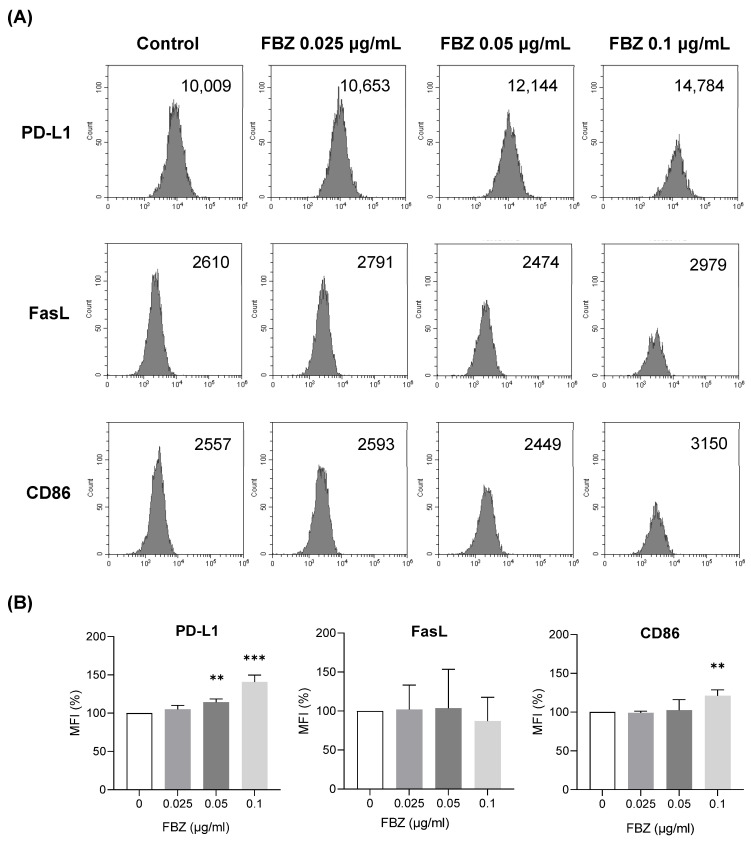
FBZ increased the expressions of PD-L1 and CD86 but not FasL on EL-4 cells. EL-4 cells were treated with FBZ for 16 h and stained for surface markers. Stained cells were analyzed using flow cytometry. (**A**) Representative figures from four repeated experiments are shown. (**B**) For statistical analysis, MFI of each marker detected in the control EL-4 cells was set to 100%. Statistical significance was performed via one-way ANOVA, followed by Dunnett’s multiple comparisons test. ** and *** indicate *p* < 0.01 and 0.001 compared to control (0 μg/mL).

We next investigated whether the effect of FBZ on EL-4 lymphoma cells in vivo was correlated with immune activity. Flow cytometry was performed to determine how FBZ affected the ligands on cancer cells that are involved in T lymphocyte immunity in the tumor microenvironment (TME) (Figure 6A,B). PD-L1 expression increased by 14% and 41% in response to treatments with 0.05 μg/mL of FBZ and 0.1 μg/mL of FBZ, respectively, and the expression of CD86 increased by 21% in response to the treatment with 0.1 μg/mL of FBZ compared with that in the control. The FasL expression did not differ from that in the control at all the FBZ concentrations.

### 3.6. FBZ Recruited M2 Macrophages in the Tumor

**Figure 7:**
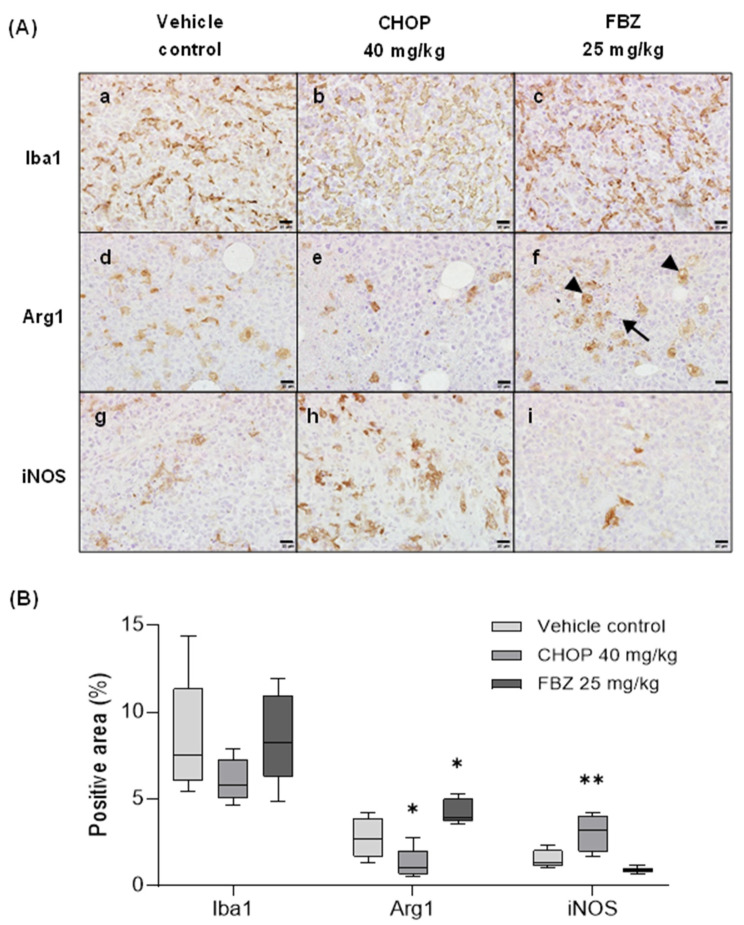
FBZ recruited M2 macrophages in tumor, while M1 macrophages were predominant in CHOP-treated tumor. (**A**) To investigate the polarized form of TAMs in tumor, IHC was performed. Tumor tissues are stained with Iba1 (**Aa**–**Ac**), Arg1 (**Ad**–**Af**), and iNOS (**Ag**–**Ai**). Apoptotic bodies (arrow) and tingible body macrophages (arrow heads) are shown in FBZ-treated tumor. IHC revealed Arg1-positive status of tingible body macrophages, forming the starry sky pattern (**Af**). Scale bar: 20 µm. (**B**) Average of the Iba-1-, Arg1-, and iNOS-positive areas of 3−5 visual fields observed at 100× per mouse is graphically shown. Statistical significance was performed via one-way ANOVA, followed by Dunnett’s multiple comparisons test. * and ** indicate *p* < 0.05 and 0.01 compared to vehicle control.

IHC was performed to determine how tumor-associated macrophages (TAMs), which are closely related to TME immunity, were altered by the FBZ treatment (Figure 7A,B). Iba-1 positivity, which indicates the total number of macrophages, did not differ between the groups (Figure 7(Aa–Ac)). Arg1 positivity, a marker of anti-inflammatory and immunosuppressive macrophages (M2), was increased by 55% in the FBZ-treated group compared with that in the control group (Figure 7(Ad,Af)). The expression of iNOS, a marker of inflammatory macrophages (M1), in the FBZ-treated group was similar to that in the control group (Figure 7(Ag,Ai)). In contrast, in the CHOP group, Arg1 was decreased by 53.5% and iNOS was increased by 99.3% compared with those of the control group (Figure 7(Ae,Ah)).

## 4. Discussion

In 2019, a patient with lung cancer in the United States experienced anticancer effects after taking FBZ, which led to a global interest in the anticancer effects of FBZ [12,20]. Since then, the anticancer effects of FBZ have been studied in vitro using various cancer cells. The anticancer effects of FBZ include the inhibition of p38-mitogen-activated protein kinase (MAPK) pathway activity and glucose metabolism in HeLa (cervical cancer) cells [11], ROS production in HL-60 (leukemia) cells [7,8], and G2/M phase arrest in 5-fluorouracil-resistant colon cancer cells SNU-C5 [10]. FBZ reportedly inhibits the growth of actively growing H4IIE (hepatocellular carcinoma) cells via inducing p21-mediated G1/S and G2/M phase arrest; however, the MAPK pathway, glucose metabolism, and ROS production were not the deemed the main mechanisms [9]. Overall, the anticancer effects of FBZ in most cancers, including lymphoma, have been poorly studied in vivo.

In this study, we investigated the anticancer effects of FBZ on EL-4 lymphoma cells in vitro and in vivo. CHOP, the first-line chemotherapy for lymphoma, was used as a positive control [16]. Cell death and decreased metabolic activity were observed in EL-4 cells treated with FBZ and CHOP (Figure 1 and Figure 2). Our results revealed that FBZ caused cell death through G2/M phase arrest, and CHOP caused cell death through ROS production (Figure 3). This mechanism is related to the antiparasitic effect of FBZ, which binds to β-tubulin in the microtubules of parasitic cells and inhibits microtubule formation [22]. 

FBZ is effectively used to treat intestinal parasites in dogs; moreover, it is poorly absorbed by the body, resulting in a long retention in the gastrointestinal tract and low plasma concentrations [23]. In this study, FBZ was administered IP because it must be absorbed systemically for the effective treatment of cancers outside of the gastrointestinal tract. Previous studies have used FBZ at 150 ppm (with diet) [24,25], 1 mg/mouse when administered orally [22], and 50 mg/kg when administered IP [26]. In the present study, the tumor size increased following the treatment with 40 mg/kg of FBZ (Figure S1A); thus, we adjusted the dose to 25 mg/kg.

In contrast to the anticancer effect of FBZ in vitro, 25 mg/kg of FBZ did not exert anticancer effects in the 7−10-week-old EL-4-bearing mice, as evident by the tumor growth being comparable to that in the control group (Figure 4 and Figure 5). Similar results were obtained when 25 mg/kg of FBZ was administered to 17−21-week-old mice; the administration of 40 mg/kg of FBZ in 7−10-week-old mice led to an increase in the tumor weight compared with the control group (Figure S1). Although there was no significant difference in the tumor volume between the control and FBZ-treated groups, the body weights of the FBZ-treated group were 4.3%, 4.8%, and 5.9% lower than those of the control group on days 4, 5, and 10, respectively (Figure 4). Notably, by day 6, the body weight of the FBZ-treated group was lower than the average weight of the CHOP-treated group, and the tumor volume showed little change. In addition, on day 10, the FBZ-treated group had the largest mean tumor volume and weight, but their body weights were lower than those of the control group. In mice, a body weight change is one of the main indicators of their health status [27]. Based on a ≥5% body weight loss on the day after FBZ administration (day 1) and a smaller body weight change compared with the other treatment groups, combined with an increase in the tumor size, it can be concluded that the FBZ-treated group experienced systemic weakness.

In vitro observations do not always reflect in vivo immune responses. Previous in vivo studies have reported the anticancer effects of FBZ in SCID [25] and nude mice [22,28,29]. A study using mice with normal immunity reported no anticancer effects of FBZ [24,26]. In the present study, the immune function of the tumor model was maintained by transplanting syngeneic lymphoma cells. Therefore, we hypothesized that the discrepancy between the in vitro and in vivo results was related to the immune activity in the body, and we further studied the effect of FBZ on immune-related factors in the TME. PD-L1 (CD274) and CD86 (B7-2) are predominantly expressed on tumor cells or antigen-presenting cells in the TME [30,31]. PD-L1 inhibits the immune responses of T cells against cancer cells by binding to PD-1 on T cells in the TME and sending an inhibitory signal to T cells. CD86 binds to CD28 on T cells to activate T cells, and it also binds to cytotoxic T lymphocyte antigen-4 (CTLA-4) on T cells to inhibit their activities. FBZ increased the expressions of PD-L1 and CD86 in EL-4 cells (Figure 6), suggesting that FBZ may contribute to the suppression of TME immunity by inhibiting T cell activity. In fact, the patient who experienced anticancer effects of FBZ also participated in a clinical trial of the PD-1 inhibitor pembrolizumab (Keytruda^®^) while receiving FBZ; therefore, his anticancer response may not have been significantly influenced by PD-L1 expression [12]. Some researchers also attributed the anticancer effects observed in this patient to pembrolizumab.

TME immunity is achieved by innate and adaptive immune cells, including T cells, dendritic cells, and macrophages [32]. Macrophages can be categorized into two states based on their polarization: M1 and M2 macrophages. M1 macrophages are stimulated by Th1 cells to produce ROS and secrete pro-inflammatory cytokines, which play important roles in innate immunity and tumor cell killing. Conversely, M2 macrophages are activated upon Th2 stimulation and secrete anti-inflammatory and immunosuppressive cytokines. TAMs belong to the M2 macrophage type. As shown in Figure 7, although there was no difference in the overall percentage of macrophages in each group, the polarization ratios of M1 and M2 were clearly different. The CHOP-treated group, wherein tumor growth was inhibited by chemotherapy, had a higher percentage of M1 macrophages, whereas the control and FBZ-treated groups, wherein tumor growth was not inhibited, had higher percentages of M2 macrophages. In particular, the FBZ-treated group showed a higher proportion of M2 macrophages than the control group, indicating that FBZ caused the immunosuppression of the TME via TAMs.

In addition to IHC, the starry sky pattern observed in the H&E-stained images suggested the presence of TAMs. The starry sky pattern refers to a histologic appearance wherein tingible body macrophages with abundant, bright cytoplasms (‘starry’) are located between strongly basophilic, darkly stained cells (‘sky’) with a high nucleus-to-cytoplasm ratio (NC ratio). This pattern is associated with apoptosis that accompanies rapid tumor growth and is mainly observed in hematolymphoid tumors or aggressive cancers with high cell proliferation rates and active apoptosis [33]. Unlike the other groups, a distinct starry sky pattern was observed in the FBZ-treated group (6/7), and these cells were Arg1-positive (Figure 5(Af) and Figure 7(Af)). Additionally, active apoptosis was observed around the periphery of this pattern (Figure 7(Af)). The accumulation of TAMs is promoted by tumor repair, nutrition, angiogenesis, tissue remodeling, and anti-inflammatory signals, and apoptotic tumor cells help to induce these signals, which, in turn, promote tumor cell proliferation [34]. In other words, our results are consistent with those of other studies showing that active apoptosis induces a starry sky pattern, indicating a high proliferation rate of the tumor.

In conclusion, FBZ showed in vitro anticancer effects by causing G2/M phase arrest. However, in a mouse model of EL-4 cell-derived lymphoma, these anticancer effects were not observed, as evidenced by the increased proliferation of cancer cells in vivo. One of the reasons for these results is the FBZ-induced alteration of immune activity in vivo. Our results indicate the onset of systemic weakness following FBZ administration; however, the cause is unknown. FBZ is also a hepatotoxic drug [35]. Further studies are required to determine the side effects of systemic FBZ and the mechanisms by which it affects the immune activity of the TME. Finally, caution should be exercised in the clinical use of FBZ due to the potential risks associated with its anticancer effects.

## Figures and Tables

**Table 1:** Primary antibodies for IHC staining.

Primary Ab	Company	Origin	Dilution
Proliferating cell nuclear antigen (PCNA)	Santa Cruz Biotechnology, Santa Cruz, CA, USA	Rabbit (polyclonal)	1:400
Ionized calcium-binding adaptor molecule 1 (Iba 1)	FUJIFILM Wako Pure Chemical Co., Tokyo, Japan		1:1000
Arginase 1 (Arg 1)	Santa Cruz Biotechnology, Santa Cruz, CA, USA		1:200
Inducible nitric oxide synthase (iNOS)	Abcam, Cambridge, UK		1:800

## Data Availability

Data are contained within the article.

## Supplementary Materials

The following supporting information can be downloaded at https://www.mdpi.com/article/10.3390/cimb45110560/s1, Figure S1: FBZ does not have anticancer effects in two different experimental conditions in vivo.
