# Supplementary material for: Fenbendazole Exhibits Differential Anticancer Effects In Vitro and In Vivo in Models of Mouse Lymphoma
Source: Curr Issues Mol Biol. 2023 Nov 8;45(11):8925–38. doi: 10.3390/cimb45110560 (PMC10670425; doi:10.3390/cimb45110560)
Supplement: Supplementary file 1 [file cimb-45-00560-s001.zip › cimb-2690595-supplementary.pdf]

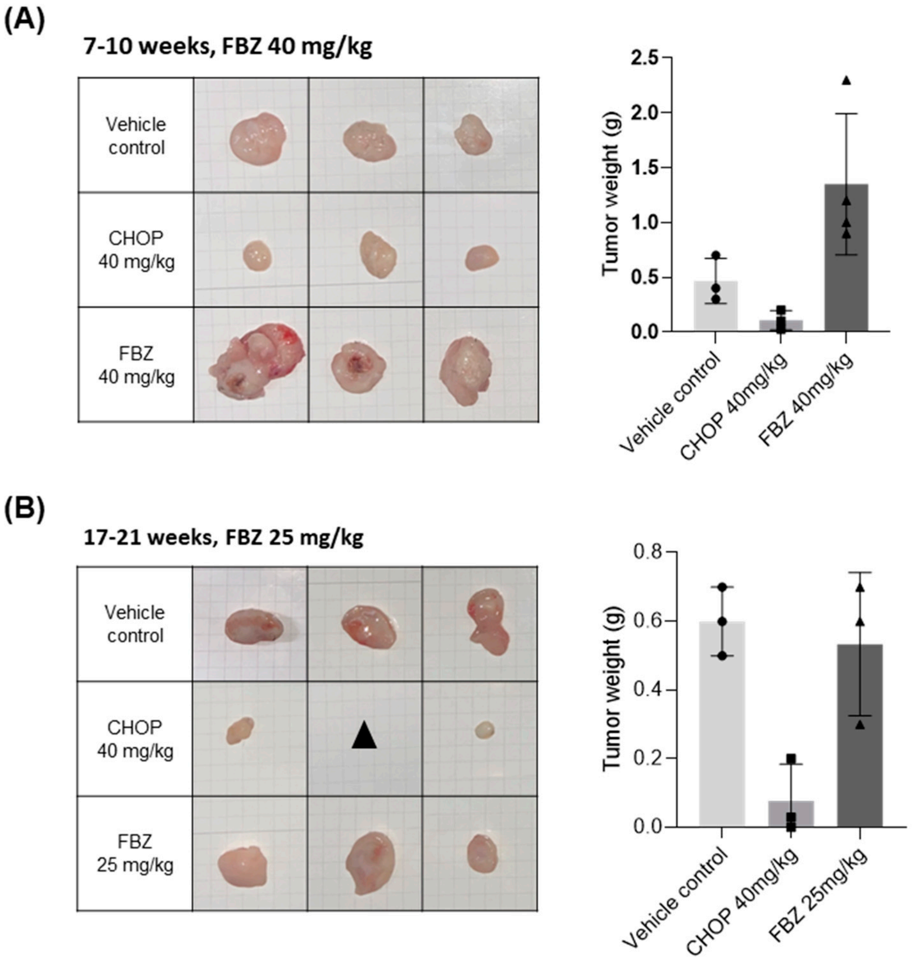

Figure S1. FBZ does not have anticancer effects in two different experimental conditions in vivo. Different doses of FBZ or different ages of mice were used to confirm that FBZ does not have anticancer effects: (**A**) 7–10-week-old C57BL/6 mice and 40 mg/kg FBZ. (**B**) 17–21-week-old C57BL/6 mice and 25 mg/kg FBZ. All drugs were injected IP, and the doses for vehicle control and CHOP-treated group are the same as described in 'Materials & Methods'. ▲: regression.
